# Diagnostic challenges and successful organ‐preserving therapy in a case of secretory carcinoma of minor salivary glands

**DOI:** 10.1002/cnr2.1491

**Published:** 2021-07-07

**Authors:** Ruben Bill, Daniel G. Deschler, Mikael J. Pittet, Sara I. Pai, Peter M. Sadow, Jong Chul Park

**Affiliations:** ^1^ Center for Systems Biology Massachusetts General Hospital and Harvard Medical School Boston Massachusetts USA; ^2^ Department of Otolaryngology ‐ Head and Neck Surgery Massachusetts Eye and Ear, Harvard Medical School Boston Massachusetts USA; ^3^ Department of Pathology and Immunology University of Geneva Geneva Switzerland; ^4^ Department of Oncology Geneva University Hospitals Geneva Switzerland; ^5^ Department of Surgery, Division of Oncologic Surgery Massachusetts General Hospital and Harvard Medical School Boston Massachusetts USA; ^6^ Departments of Pathology Massachusetts General Hospital and Harvard Medical School Boston Massachusetts USA; ^7^ Department of Medicine Massachusetts General Hospital and Harvard Medical School Boston Massachusetts USA

**Keywords:** head and neck cancer, larotrectinib, minor salivary glands, *NTRK* fusion, secretory carcinoma

## Abstract

**Background:**

Secretory carcinoma is a more recently described subtype of salivary gland carcinoma that may pose diagnostic challenges and frequently harbors *NTRK* fusions that may successfully be targeted by TRK inhibitors in advanced disease.

**Case:**

We present the case of a female patient with secretory carcinoma arising in the base of tongue with persistent disease after debulking surgery and definitive chemoradiation. As an alternative to salvage surgery, which would have resulted in significant impairment of swallowing and speech function, a targeted therapy with the TRK‐inhibitor larotrectinib against an identified *ETV6‐NTRK3* fusion product was initiated. Larotrectinib treatment has been well tolerated, resulted in durable complete response and the patient maintains good swallowing and speech function.

**Conclusion:**

The presented case underscores the importance of the accurate diagnosis of secretory carcinoma. It further highlights the impact of molecular testing as targeted therapies may play an important role in the management of advanced salivary gland cancers.

## 1 INTRODUCTION

Secretory carcinomas (SC) of the salivary gland were classified over a decade ago, initially thought to be a more aggressive subtype of acinic cell carcinoma that bore some homology to secretory carcinomas of the breast. Initially classified as mammary analogue secretory carcinomas (MASC),^1^ the term was revised in the most recent edition of the WHO classification of Head and Neck Tumors (2017).^2^ In addition, the initial characteristic t(12,15) (p13;q25) translocation resulting in the *ETV6‐NTRK3* fusion transcript has been noted in these tumors, and more recently, occurring with some regularity associated with *RET* fusions.^3^, ^4^ This tumor typically occurs in the major salivary glands; however, SC can also occur in atypical locations arising in minor salivary glands.^5^, ^6^ The histopathologic diagnosis of SC may be challenging, although it is more commonly recognized since its classification. Initially identified as an aggressive subset of acinic cell carcinomas, it is now recognized to be a more indolent tumor, but with a spectrum of clinical behavior. Although most SC may develop over years, cases with an aggressive disease course, including lymph node metastases along with local recurrence or even distant metastases with lethal outcomes have been reported.^1^, ^7^ The rare occurrence of high‐grade transformation in SC of major and minor salivary glands is often associated with an unfavorable prognosis.^8^

Curative treatment strategies for SC of the salivary glands are identical to those of other salivary gland tumors concentrating on complete surgical resection. Dependent upon tumor stage and specific pathological features such as grade, perineural‐ and lymphovascular invasion and margin status, additional therapeutic modalities are considered, including adjuvant radiotherapy or chemoradiation. In the case of local recurrence or persistent disease of previously irradiated tumors, surgery, reirradiation or chemoradiation are the therapies of choice.^9^ Based on the frequency of fusion kinases associated with these tumors, nearly all, they are ideal candidates for targeted therapy,^1^, ^3^, ^4^, ^7^ especially in the setting of advanced, recurrent or palliative disease. With the dominant *ETV6*‐*NTRK3* fusion, this tumor entity appears as an ideal candidate for inhibitors targeting tropomyosin receptor kinase (TRK) proteins, like the small‐molecules larotrectinib or entrectinib. Indeed, larotrectinib and entrectinib therapy both induced rapid and durable relevant clinical activity in *NTRK* fusion‐positive solid tumors including cases of salivary SC with locally advanced or metastatic disease.^10^, ^11^, ^12^

In this case report, we discuss the challenges of the proper diagnosis of SC, and the use of larotrectinib in a potentially salvageable recurrent tumor that would otherwise had exceptionally debilitating outcomes if surgically resected.

## 2 CASE PRESENTATION

A 56‐year‐old White female was referred to her local otolaryngologist for further evaluation of a chronic sore throat lasting several months that failed conservative management. The otherwise healthy woman reported a personal history of 10 pack years of cigarette smoking until about 35 years prior and continued moderate alcohol intake (3–4 drinks per week). Laryngoscopy showed a mass likely originating from the base of tongue (BOT) that was biopsied and determined to be malignant. Histopathologic work up including immunohistochemical analysis revealing diffuse positivity for cytokeratin 7, weak, non‐specific staining for GATA3 and estrogen receptor, and negative staining for PAX‐8, CDX‐2, TTF1, p40 and cytokeratin 5/6. Together with the morphological appearance, the diagnosis of an adenocarcinoma with papillary features was made. Staging by positron emission tomography (PET)‐ computed tomography (CT) scan confirmed a fluorodeoxyglucose (FDG)‐avid mass involving the left and mid BOT and an FDG‐avid enlarged lymph node in the right neck (2.0 × 1.5 cm) and two additional subcentimeter lymph nodes in the left neck with faint FDG‐avidity, suggestive of regional metastatic disease, but with no indication of distant metastases. The patient was diagnosed with stage cT2N2cM0 disease (AJCC UICC 8th edition). Subsequently, the patient underwent debulking surgery of the BOT mass followed by definitive chemoradiation with carboplatin/paclitaxel weekly and radiotherapy with a cumulative dose of 70 Gy in 35 fractions. A follow‐up PET‐CT scan 3.5 months after completion of chemoradiation indicated a reduction in size of both the supraglottic mass and cervical lymph nodes. However, the FDG‐avidity continued to be in the range of possible persistent disease. Consequently, re‐biopsy of the residual mass of the BOT was performed with a subsequent pathological diagnosis of a p16‐positive squamous cell carcinoma (SCC). Due to the discrepant pathological findings, the patient's biopsies were sent from the originating hospital for pathologic consultation. Comparable morphology between the primary tumor and the persistent lesion suggested a poorly differentiated carcinoma favoring adenocarcinoma as the most likely diagnosis. The p16 staining was assessed by the pathology consultant as focal, non‐specific staining.

Given the complexity of this case, the patient subsequently referred to our tertiary care center for further evaluation and discussion of therapeutic options.

**FIGURE 1:**
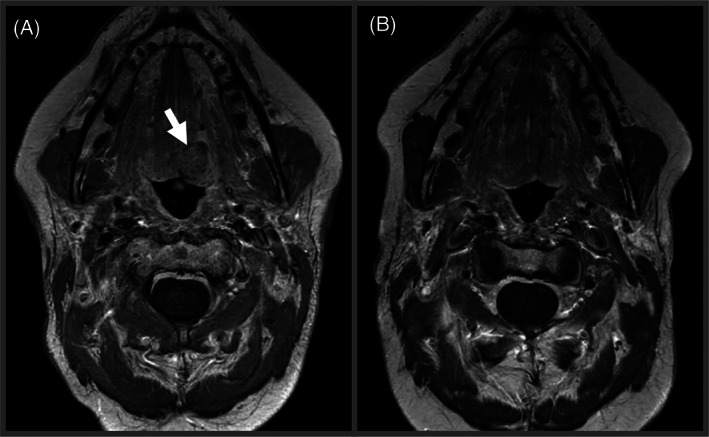
Treatment with larotrectinib resulted in complete resolution of the persistent secretory carcinoma of the left BOT (A, arrow) as illustrated with representative T1 TSE MRI images (3 mm slices) before initiation of larotrectinib therapy (A) and in the most recent imaging study after 11 months on therapy (B)

**FIGURE 2:**
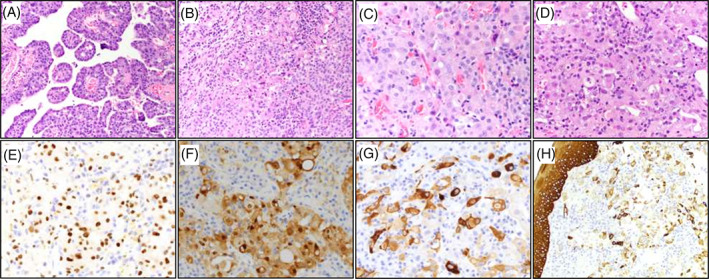
Hematoxylin and Eosin stains of the patient's initial biopsy of the primary tumor (A and B; 200X magnification), the persistent lesion of the BOT 6 months later (C; 400X) and of a second biopsy of the same persistent tumor taken in the midline vallecula region an additional 3 weeks later (D; 400X). Panel (A) shows focal papillary architecture with fibrovascular cores often associated with secretory carcinomas and tumor cells contain enlarged, hyperchromatic nuclei. The three sequential biopsies shown in panels (A–D) are consistent with morphologic features of secretory carcinoma: sheet‐like morphology with foci of cytoplasmic clearing and pink (eosinophilic) cytoplasm with variably dense nuclei showing conspicuous nucleoli. Immunohistochemistry of the midline vallecular biopsy of the persistent tumor shows positive nuclear staining in tumor cells for GATA 3 (E, 400X), and cytoplasmic staining for mammaglobin (F, 400X) and S100 (G, 400X). Panel H shows intact surface epithelial staining (left) for squamous/myoepithelial marker keratin 5/6 with the tumor (right) showing multifocal positivity (200X). The immunohistochemical findings support a diagnosis of high‐grade secretory carcinoma (with supporting molecular features of an *ETV6‐NTRK3* fusion gene)

Consistent with the previous examinations, magnetic resonance imaging (MRI) showed a heterogenous, lobulated lesion in the left BOT (Figure 1A) as well as bilateral abnormal lymph nodes in levels 2A (right) and levels 3B and 4 (left). Three weeks after the biopsy that diagnosed p16+ SCC, an additional biopsy in the area of the midline vallecula was performed at our hospital. Histopathological analysis of the biopsy specimen showed an – at least in the assessed area – entirely high‐grade epithelioid malignant neoplasm with papillary and microcystic architecture, extensive focal necrosis, large round nuclei with prominent nucleoli, focally a vacuolated cytoplasm and eosinophilic secretions, morphologically comparable to the primary tumor and first biopsy of the persistent lesion performed at the outside hospital (Figure 2A–D). Immunohistochemistry revealed diffusely positive staining for GATA3, mammaglobin, S‐100, keratin 5/6 (Figures 2E–H), and keratin 7. Tumor cells were negative for p40, ERG, desmin and smooth muscle actin. The case was diagnosed as a high‐grade secretory carcinoma with molecular testing requested. This subsequently showed a confirmatory *ETV6‐NTRK3* fusion transcript involving *ETV6* exon 5 and *NTRK3* exon 15. Potential treatment options were discussed with the patient, including the standard treatment involving salvage surgery. Given that salvage surgery would be quite morbid including laryngectomy and glossectomy with for the patient significant functional impact, alternative therapy with an TRK‐inhibitor was discussed as a potential function‐preserving treatment strategy. The patient proceeded with the small molecule TRK‐inhibitor larotrectinib. Larotrectinib was initiated and was administered at a dose of 100 mg BID. Although an initial radiographic assessment following 1.5 months on larotrectinib therapy showed no significant decline in tumor burden, following 4.5 months of treatment, a complete clinical response was seen by CT scan. In addition, larotrectinib was very well tolerated by the patient with no adverse serological features. The patient's speech was well‐maintained with no difficulty swallowing. At the time of this report, the patient has been on continuous Larotrectinib for 13 months, currently with a maintained complete response (Figure 1B). She continues on this therapy with regular clinical and radiographic follow up visits until the potential occurrence of evasive resistance.

## 3 DISCUSSION

As in the presented case, misclassification of salivary gland tumors is not uncommon, especially with small biopsies or when tumors present in atypical sites.^7^, ^13^ Most commonly, SC have been traditionally associated with acinic cell carcinoma (AciCC), as they have some overlapping morphological and architectural features, including a microcystic growth pattern, solid sheets of cells and micropapillae, but less so as pathologists become more familiar with its diagnosis. In contrast to most AciCC, typically diffusely positive for DOG1 (discovered on GIST‐1) by immunohistochemistry, SC are typically diffusely positive for S‐100, mammaglobin, and GATA3 and negative for DOG1. In addition, the detection of a fusion kinase, *ETV6‐NTRK3* in most SC of salivary glands and *ETV6‐RET* in a significant subset, confirms the diagnosis.^1^, ^4^ This characteristic chromosomal translocation can be detected by reverse transcription‐polymerase chain reaction (RT‐PCR), targeted fusion transcript detection by next generation sequencing or fluorescence in situ hybridization (FISH).^14^, ^15^ In routine clinical settings without the option for molecular evaluation, diagnosis will need to focus on strict morphological criteria including a limited immunohistochemical panel if possible. Use of a pan‐TRK antibody for immunohistochemistry can provide some aid in the differential diagnosis of SC versus AciCC, but the antibody, although specific, is not particularly sensitive,^16^ and given additional fusion partners, an initial screen would benefit more from DOG1/S100 in the differential, raising follow up testing once a diagnosis of SC is suspected, especially in any case of advanced disease or when the patient is a poor surgical candidate for other reasons. And, as described, although the *ETV6‐NTRK3* fusion represents the most common genomic aberration in SC of salivary glands, there are oncogenic rearrangements of *ETV6* with fusion partners other than *NTRK3*, such as *MAML3* or *RET*, and cases without *ETV6* fusions have been described.^7^, ^17^

Whereas in the curative stage, surgery with appropriate adjuvant treatment represents the best treatment modality, in the case of unresectable or metastatic disease, targeted therapy with TRK‐inhibitors represents a highly active therapeutic opportunity. In a pooled analysis of phase I and phase II clinical trials of solid tumors with TRK‐fusions, 79% and 16% of the patients experienced an objective response and complete response, respectively, upon larotrectinib treatment at a dose of 100 mg BID as administered in the presented case.^12^ In patients that responded to the treatment, the median duration of response was 35.2 months.^12^ As the development of resistance to TRK‐inhibitors is likely, a continuous clinical and radiographic follow up is important. Both, on‐ and off‐target resistance mechanisms to TRK inhibitors in solid TRK‐fusion positive tumors have been reported. On‐target mutations affect the TRK‐kinase domain and thus restrict drug binding, and can potentially be circumvented by second‐generation TRK inhibitors such as LOXO‐195 that is currently evaluated in a clinical trial including *NTRK* fusion positive solid tumors with intrinsic or evasive resistance to a prior TRK inhibitor (NCT03215511).^18^ In contrast, off‐target mutations typically cause resistance to second‐generation TRK inhibitors and can occur during treatment with first‐ and second‐generation TRK inhibitors. Different off‐target genomic alterations that cause activation of the mitogen‐activated protein kinase (MAPK) pathway or MET amplification have been reported.^19^ Thus, in the presented case, in the event of evasive resistance, a biopsy of the emerging lesion will be critical to determine the molecular mechanism of resistance. Based upon updated genetic alteration data, a follow up treatment strategy will be employed, including further targeted therapies or salvage surgery.

The era of targeted therapies in oncology opens avenues for creative treatment approaches in selected clinical situations. In the presented case, salvage surgery as the standard treatment option of this persistent SC of minor salivary glands would have been technically feasible, yet likely led to significant impairment of the patient's quality of life. As the response rate of TRK‐inhibitors in TRK‐fusion positive solid tumors is substantially high, a treatment attempt to either downstage the tumor in the case of a partial response to facilitate surgery, or, as in the presented case, due to the complete response that will significantly delay repeat surgery until the likely recurrence of evasive resistance is quite appealing, and as shown in this case, quite successful.

## CONFLICT OF INTEREST

M.J.P. has served as a consultant for Aileron Therapeutics, AstraZeneca, Cygnal Therapeutics, Elstar Therapeutics, ImmuneOncia, KSQ Therapeutics, Merck, Siamab Therapeutics, Third Rock Ventures. The wife of R.B. is an employee and stockholder of CSL Behring. These commercial relationships are unrelated to the current study.

## AUTHOR CONTRIBUTIONS

*Conceptualization; data curation; investigation; project administration; resources; visualization; writing‐original draft; writing‐review & editing*, R.B.; *Data curation; resources; writing‐review & editing*, D.D.; *Supervision; visualization; writing‐review & editing*, M.P.; *Conceptualization; data curation; writing‐review & editing*, S.P.; *Conceptualization; data curation; resources; supervision; visualization; writing‐review & editing*, P.S.; Conceptualization; data curation; project administration; resources; supervision; visualization; writing‐original draft; writing‐review & editing, J.C.P.

## ETHICS STATEMENT

Written informed consent was obtained from the patient. The institutional approval was waived by the Institutional Review Board for this single patient case report.

## Data Availability

The data that support the findings of this study are available on request from the corresponding author. The data are not publicly available due to privacy or ethical restrictions.
